# Adoption of Durum Wheat Cultivar ‘Salim’ with a Technical Package and Its Resilience to Climate Change Impacts in Smallholders: Case of Nebeur/Kef Region, Tunisia

**DOI:** 10.3390/plants10112379

**Published:** 2021-11-05

**Authors:** Sourour Ayed, Saida Mlouhi, Imen Bouhaouel

**Affiliations:** 1 Field Crops Laboratory, LR20-INRAT-02, National Agricultural Research Institute of Tunisia, University of Carthage, Ariana 2049, Tunisia; 2 Rural Economy Laboratory, LR20-INRAT-07, National Agricultural Research Institute of Tunisia, University of Carthage, Ariana 2049, Tunisia; saidamlouhi@gmail.com; 3 Genetics and Cereal Breeding Laboratory, LR14AGR01, National Agronomic Institute of Tunisia, University of Carthage, Tunis 1082, Tunisia; imenbouhaouel@gmail.com

**Keywords:** CropSyst model, mitigation, semi-arid environment, simulation technical package, sustainability

## Abstract

In recent years, there has been an urgent need for local strategies to ensure food sustainability in Tunisia, recognized as a climate change hotspot region. In this context, adaptation measures, including the adoption of high-yielding durum wheat cultivars with adequate agronomical practices, are an important avenue to improving the productivity of the smallholders that represent 80% of Tunisian farmers. Thus, this study highlights the impact of (i) the adoption of the recently marketed durum wheat cultivar ‘Salim’ as compared to the common cultivar ‘Karim’ and the transfer of a technical package to 11 farmers in the Nebeur delegation/Kef-Tunisia (semi-arid region) during the 2013/2014 and 2014/2015 cropping seasons, and (ii) climate change on the expected mean grain yield and biomass by 2070, using the CropSyst agronomic cultivation model based on multi-year crop simulations run with a daily weather series (2020–2070). The adoption of ‘Salim’ with the recommended package, compared to ‘Karim’ with the farmer practices, significantly increased the grain yield (37.84%) and biomass (55.43%). Otherwise, the impact of the 0.8 °C temperature rise on the potential yields and biomass over the next 51 years was positive. Contrary to expectations, the yield increases for the two cultivars were very close, but the yield of ‘Salim’ (36.02 q ha^−1^) remains much higher than that of ‘Karim’ (23.34 q ha^−1^). On other hand, ‘Salim’ experienced a higher increase for biomass compared to that of ‘Karim’. These results indicate that the adoption of the ‘Salim’ cultivar with its technical package might be considered as a strategy of adaptation to Nebeur conditions and to future climate change events.

## 1. Introduction

In recent years, durum wheat (*Triticum durum* Desf.) production has been threatened by climate change and the extreme weather events that pose a serious risk to social, economic, and political stability in the Mediterranean countries, particularly in Tunisia [1,2,3]. In fact, durum wheat is the staple food that provides the main import of calories (258 kg/capita/year) in this country [3,4]. It is well known that the response of wheat to climate change varies between cultivars, years, pedological conditions, and agronomic management, thus highlighting the need to consider all these factors in climate change impact studies [3]. The influences of weather and climate on the different components of crop production can vary and often happen at the same time [5]. Furthermore, different types of climatic extremes can affect the crop production differently. This makes it difficult to understand the climatic impacts on the respective components of crop production. The increase in temperature in response to climate change raises the risk of heat stress and water demand during flowering time and boosts earlier and faster crop growth, thereby reducing yield [6,7]. On the other hand, higher temperature can reduce the risk of severe/late frost damage and thus leads to higher yield and biomass accumulation.

Stabilizing and increasing production under these conditions will be a real challenge for Tunisia, requiring an improvement in technical and economic efficiencies [8]. Accordingly, adaptation strategies that are resilient to such changes across different climates should be implemented immediately, and the government must take decisive action to ensure sustainable food production. From these measures, crop genetics and management strategies, such as technological advances, certified seeds and fertilizers, the adoption of new cultivar, and better farming practices, can be options to increase production by the smallholders that represent 80% of Tunisian farmers [9,10,11]. Several regions already showed large yield gaps caused by the lack of some of these components [12]. To boost the productivity of Tunisian farmers, especially in rainfed environments, great efforts were made by the national breeding program, resulting in the registering of several durum wheat cultivars in the national catalogue (e.g., ‘Karim’, ‘Razzek’, ‘Om Rabiaa’, ‘Khiar’, ‘Nasr’, ‘Maali’, and ‘Salim’) [13,14]. In particular, increasing yield genetic potential under water-deficit conditions is one of the major objectives and the principal selection index of the durum wheat breeding programs [3,15,16]. Promoting the adoption of high-yielding cultivars with the recommended practices in a sustainable manner helps to improve the livelihoods of rural farmers [17]. In particular, on-farm demonstrations, which are key to teaching in the extension system, gained the confidence of farmers who toured the farms, leading to the successful adoption of the package and the showing of new practices to the farmers [18]. Accordingly, there is a need for adoption studies to consider the farmers’ perceptions of the technology attributes in the evaluation of the durum wheat cultivar adoption decisions.

According to Lobell et al. [19], the way to better understand the impacts of climate change on yields and to develop effective adaptation strategies is to assess the extent to which historical and recent crop yield trends have been affected by climate change. Several statistical models were used as reliable tools for the quantitative prediction of crop yield [20]. These crop-yield simulation models integrate knowledge on physiology, agronomy, soil science, and agro-meteorology in the models, using mathematical equations to quantitatively and dynamically describe the process of crop growth, development, and yield establishment [21,22,23]. Several studies have attempted to relate the climate to crop yields, with or without considering the technological advances at both the regional and the global scales [24]. A recent study predicted a strong decline in global wheat yields by 6.0 ± 2.9% for each 1 °C of temperature rise due to climate change [25]. The estimated wheat-yield losses for the United States (−5.5 ± 4.4% per 1 °C) and France (−6.0 ± 4.2% per 1 °C) are similar to the global average, while those for India (−9.1 ± 5.4% per 1 °C) and Russia (−7.8 ± 6.3% per 1 °C) are more vulnerable to temperature increase. Otherwise, Bahri et al. [8] reported that the durum wheat grain yield will increase differently in the semi-arid region (Kef region) according to agronomic scenarios for the considered time slices (2016–2035, 2045–2064, and 2075–2094). Taking all of these factors into account, the study of the degrees of effectiveness of the adaptation strategies in the future according to local conditions is required to obtain climate-smart and sustainable food systems.

Thus, the present investigation was designed to promote a mitigation strategy by (i) the adoption of ‘Salim’ cultivar, one of the recently marketed Tunisian durum wheat cultivars, chosen mainly for its yield potential and drought tolerance, and (ii) the transfer of the durum wheat technical package to the smallholders of the Nebeur/Kef region-Tunisia, located in the semi-arid area. The second aim was to analyze the current climate status and future projected climate changes on the adaptation of this cultivar with its recommended package from 2020 to 2070. In this study, the agronomic cultivation model, CropSyst, was selected to predict the grain yield and biomass over the next 51 years.

## 2. Results and Discussion

### 2.1. On-Farm Demonstration of ‘Salim’ Durum Wheat Cultivar

**Figure 1:**
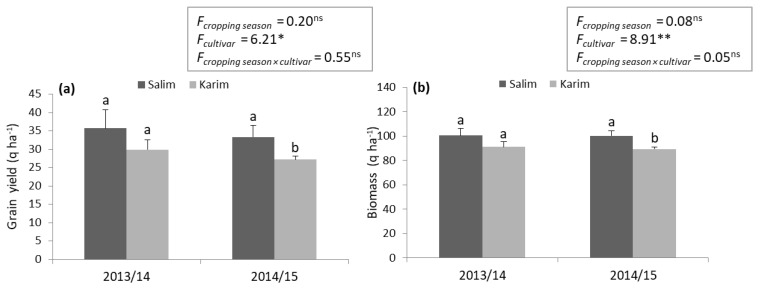
Effect of ‘Salim’ cultivar adoption based on mean grain yield (**a**) and biomass (**b**), compared to ‘Karim’ cultivar using the recommended technical package. Values represent the mean ± SD and different letters indicate significant differences between cultivars at *p* ≤ 0.05 (Tukey test) for each cropping season. *F*: *F* value; ns: non-significant; *: significant at the level 0.05; **: significant at the level 0.01.

As a first step, on-farm cultivar testing aims to assess the performance of the emerging and current cultivar ‘Salim’ in large plots as compared to that of the common cultivar ‘Karim’, using the same recommended technical package by all farmers (i.e., leader and satellites). In Tunisia, most farmers used the old cultivar ‘Karim’, characterized by its sensitivity to diseases (e.g., *Septoria tritici* blotch) (Table S2) [26], with the traditional agricultural practices, while only a few adopted some of the new durum wheat cultivars. Analysis of variance (ANOVA) showed significant differences in grain yield (*p* < 0.05) and biomass (*p* < 0.01) between ‘Salim’ and ‘Karim’ for the two cropping seasons, 2013/14 and 2014/15 (Figure 1). The highest mean grain yield and biomass were recorded for ‘Salim’ (34.52 and 100.19 q ha^−1^, respectively) compared to ‘Karim’ (28.55 and 90.23 q ha^−1^, respectively). Thus, the increase in terms of yield and biomass for the farmers who adopted ‘Salim’ cultivar is about 17.32% and 9.93%, respectively. Our findings corroborate those of Tiwari et al. [27] and Bekele and Shiberu [28] who studied the adoption of improved wheat varieties by Indian and Ethiopian farmers. The adoption of the new cultivars depends on several factors, either directly or indirectly. Bekele and Shiberu [28] depicted that high cost and access to certified seeds, the lack of income of the farmers, and the low education level are the major constraints that negatively affect the adoption rate of the improved bread wheat varieties. In addition, farm and field characteristic variables, such as farm size, soil type, and animal power play significant roles in adoption decisions [29]. Nonetheless, the farmers’ participation in the on-farm demonstration, training course and field day on wheat production, and the farmers’ access to the extension service, might significantly affect the adoption rate of the recommended durum wheat cultivars.

### 2.2. Transfer of Durum Wheat Technical Package

**Figure 2:**
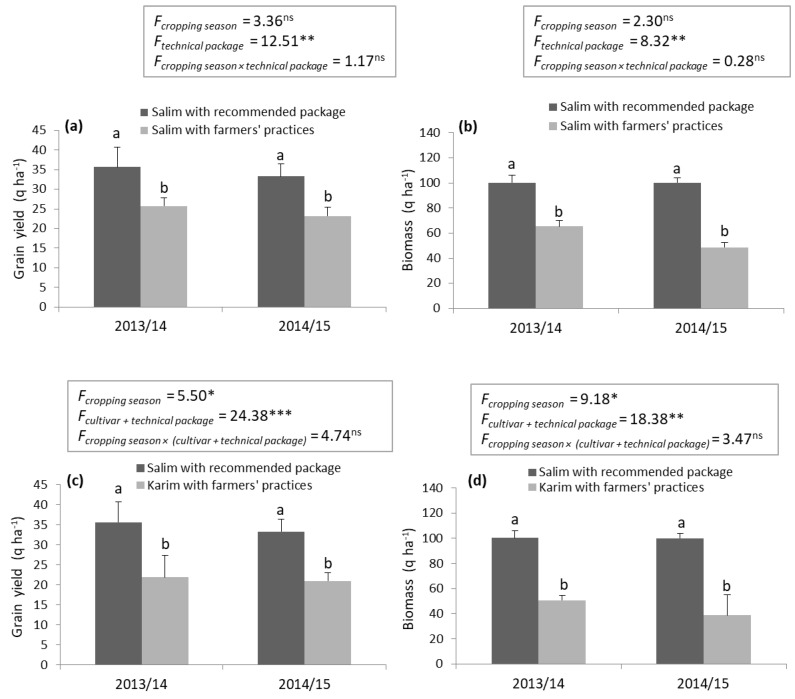
Effect of ‘Salim’ cultivar and technical package adoption based on mean grain yield (**a**,**c**) and biomass (**b**,**d**). Values represent the mean ± SD and different letters indicate significant differences between technical package at *p* ≤ 0.05 (Tukey test) for each cropping season. *F*: *F* value; ns: non-significant; *: significant at the level 0.05; **: significant at the level 0.01; ***: significant at the level 0.001.

The efficiency of the crop-production package was determined using the ‘Salim’ cultivar that had already showed better performance compared to ‘Karim’. The results revealed significant variations (*p* < 0.01) between the demonstration package and the farmers’ practices (Figure 2a,b). The mean grain yield and biomass of ‘Salim’ with the recommended package were clearly higher (34.52 and 100.19 q ha^−1^, respectively) than those obtained by the classical practices (24.46 and 56.99 q ha^−1^). For the two cropping seasons (2013/14 and 2014/15), the technical package increased the mean grain yield of ‘Salim’ by 29.17%, and the biomass by 43.12%. As expected, these results were achieved by improved management practices, including fertilizer application and the mechanization of weed, pest, and disease control. In the same context, Adzawla and Alhassan [30] found that among the climate adaptation strategies adopted by maize farmers, crop rotation and row planting were essential for sustainable food production in Northern Ghana. By comparing the farmers’ practices to the recommended practices, Joshi et al. [31] reported that the demonstrated plot gave an additional yield of 6.67 q ha^−1^ and an increase in durum wheat productivity of about 18.22%. Additionally, Tiwari et al. [27] mentioned that improved wheat technology increased yield with an average of 13.23 q ha^−1^. However, according to Eponou [32], only a small proportion of farmers (10%) adopted all the components of the technology packages in Africa. Thus, further work is needed to encourage the traditional farmers to adopt the improved durum wheat technology.

### 2.3. Adoption of ‘Salim’ with the Recommended Technical Package vs. ‘Karim’ with Farmers’ Practices

To convince the farmers to abandon their usual practices and adopt new technologies, yield and biomass increases were estimated by comparing ‘Salim’ with the recommended package vs. ‘Karim’ with the farmers’ practices. A significant difference (*p* < 0.01) was obtained between the technical itineraries for the two attributes (Figure 2c,d). Interestingly, the technical package proposed in this study (‘Salim’ cultivar, fertilizer, and weed and disease control) increased the mean grain yield and biomass by 37.84 and 55.48%, respectively.

Yield gap was calculated as the difference between the yield levels using the recommended package and the yield levels using the existing farmers’ practices. In our conditions, mean yield gaps of 13.82 and 12.36 q ha^−1^ were recorded for the 2013/14 and 2014/15 cropping seasons, respectively. These results were achieved through improved management practices, combined with the use of the new high-yielding cultivar. Although these farmers share the same agricultural practices (i.e., cultivar and technical package), differential yield gaps were observed for the considered farmers (Table S1). This variation might be attributed to some factors, such as soil type, the preceding crop, weed flora, etc. In the same sense, Joshi et al. [31] obtained mean wheat yield gaps ranging between 5.34 to 8.12 q ha^−1^ depending on the crop year. These authors suggest that the use of the improved method of wheat cultivation can reduce the technology gap (i.e., the difference between the potential yield and the demonstration yield) and increase wheat productivity. Large production increases from 45% to 70% are possible with the changing of management practices [10]. In particular, Bell et al. [33] reported that 48% of the gain was attributed to the increased use of N fertilizer. In conclusion, farmers are recommended to leave the classical practices in favor of the durum wheat package proposed by the project in order to improve their production and incomes.

### 2.4. Generated Temperature and Precipitation

**Figure 3:**
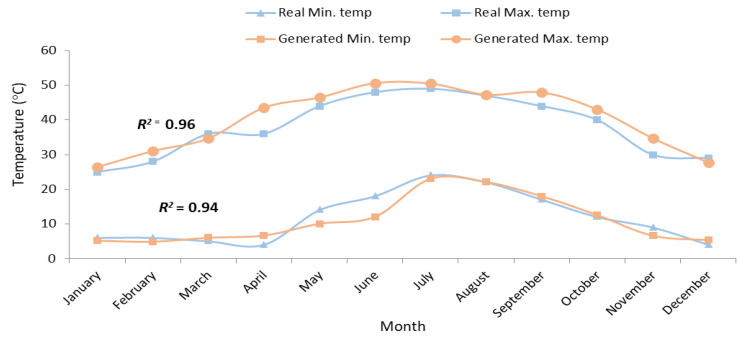
Real (1992–2019) vs. generated (2020–2070) mean temperature.

The grain yield and biomass of ‘Salim’ and ‘Karim’ cultivars were simulated based on past weather data (1992–2019) and future weather scenarios (2021–2070). The generation of temperature by the crop system showed that the real mean maximum temperature will increase from 38.0 °C during the period 1992–2019 to 40.3 °C during 2021–2070, an increase of 2.3 °C (Figure 3). On the other hand, the mean minimum temperature for the same period (1992–2019) will drop from 11.8 °C to 11.1 °C around the 2070s, i.e., a decrease of −0.7 °C (generated temperature). Comparing the real mean of maximum and minimum temperatures, a temperature rise of 0.8 °C will be recorded at the end of the period considered by the temperature projection. Tunisia is already recognized as a climate change hotspot region in the Mediterranean Rim [34]. This is due to the location of the country, positioned between the inter-tropical regions and the temperate regions of the northern hemisphere, which makes its climate particularly variable. Our results are in accordance with those of the Intended Nationally Determined Contribution (INDC), which reported that the country is very exposed to climate change, characterized by an average temperature increase of 2.1 °C by 2050 [35]. Assuming an overall temperature rise of +2 °C, Pillet et al. [36] also concluded that the annual temperature will increase from +1.5 °C to +2.8 °C from the north to the south of Tunisia during the period of 2031–2060. The seasonal increases will vary from +1.7 °C to +2.8 °C for autumn; from +1.8 °C to +3.5 °C for summer; from +1.2 °C to +2.2 °C for spring; and from +1.3 °C to +2.3 °C for winter. Otherwise, by comparing sub-humid to semi-arid areas of Tunisia, Bahri et al. [8] reported that temperature increases of 1.5 °C and 1.7 °C, respectively, will be projected over the period 2075–2094, as compared to 2016–2035.

**Figure 4:**
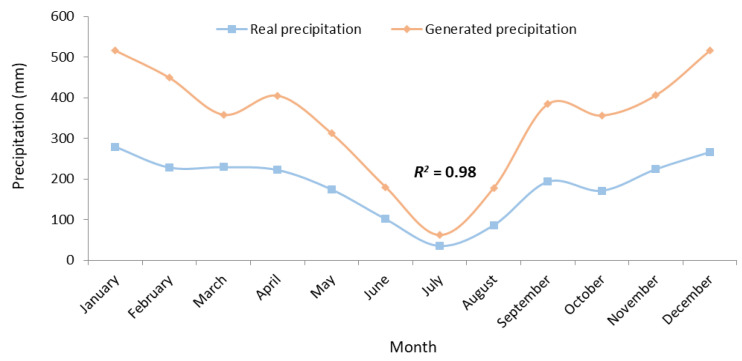
Real (1992–2019) vs. generated (2020–2070) precipitation.

Our analysis revealed that the mean of the real precipitation of 184 mm during the period 1992–2019 will increase to 344 mm by the 2070s, an increase of 160 mm (Figure 4). Easterling et al. [37] also showed changes in extreme events for future climates, such as increases in extreme high temperatures, decreases in extreme low temperatures, and increases in intense precipitation events. The same result was obtained by other climate model simulations showing that an increase in greenhouse gases produces increased surface heating with warmer surface temperatures and more evaporation [38]. This leads to an increase in the atmosphere’s ability to hold more atmospheric moisture content with enhanced precipitation rates. The IPCC [39] mentioned that changes in precipitation in a warming world will not be uniform, and extreme precipitation events will become more intense and frequent in many regions. For instance, Liu et al. [40] reported that under the 1.5 °C scenario in Central Asia, the mean annual total precipitation and heavy precipitation will experience increases of 7.68% and 26.55%. Conversely, the INDC [35] predicted for Tunisia an increase in average temperature of 2.1 °C and a 20% decrease in annual rainfall by 2050. Bahri et al. [8] also noted that the cumulative rainfall for the three time slices considered (2016–2035, 2045–2064, and 2075–2094) is not going to change in the Kef region, while it will significantly decrease in the sub-humid area.

A clearly close correspondence (*R*^2^ > 0.90) between the real and generated temperatures or precipitations (Figure 3 and Figure 4) was obtained, indicating that ClimGen (sub-program in CropSyst model) adeptly generates climate data in the Tunisian conditions.

### 2.5. Short-Term Grain Yield and Biomass Simulation

**Figure 5:**
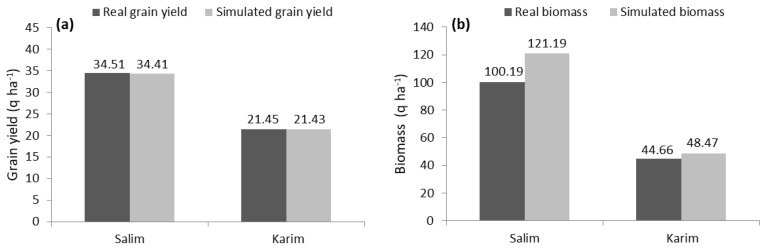
Real vs. simulated grain yield (**a**) and biomass (**b**) of ‘Salim’ and ‘Karim’ cultivars (2013/14 and 2014/15).

A comparison between the observed yields (34.51 and 21.45 q ha^−1^ for ‘Salim’ and ‘Karim’, respectively) and the simulated yields (34.41 and 21.43 q ha^−1^ for ‘Salim’ and ‘Karim’, respectively) highlights the proximity of values (Figure 5). Similar to the grain yield, the values of the real (44.66 q ha^−1^) and the simulated biomass (48.47 q ha^−1^) of ‘Karim’ cultivar were very close. However, the simulated biomass of the ‘Salim’ cultivar (121.19 q ha^−1^) was higher than the observed biomass (100.19 q ha^−1^). In reality, and taking into account natural conditions, several factors negatively affect the yields, such as losses due to diseases, handling, transport, packaging, and storage. These factors are not modeled and might explain the variation between the real and simulated biomass as confirmed by Flichman and Jacquet [41].

### 2.6. Long-Term Grain Yield and Biomass Simulation

**Figure 6:**
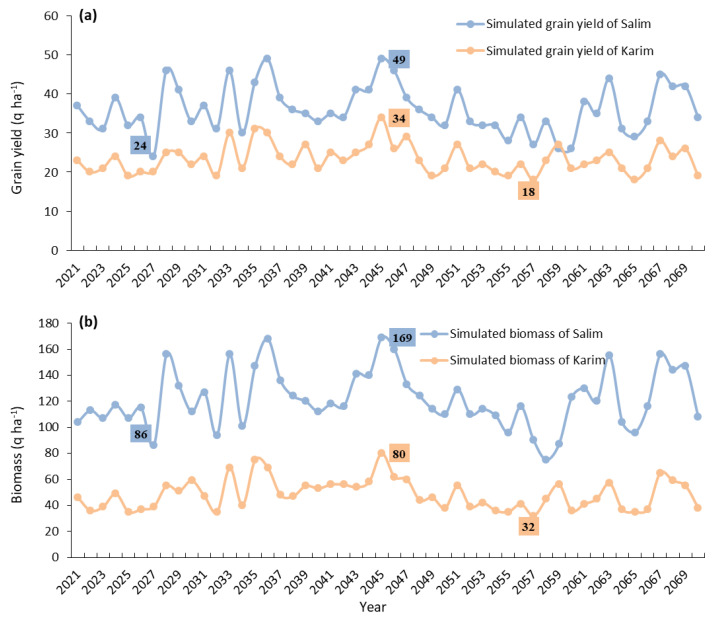
Simulated grain yield (**a**) and biomass (**b**) (2020–2070) of ‘Salim’ and ‘Karim’ cultivars.

The real yields of ‘Salim’ and ‘Karim’ were 34.51 and 21.45 q ha^−1^ (Figure 5), respectively, while the predicted yields over 2020–2070 (51 years) were 36.02 and 23.34 q ha^−1^ (Figure 6). Surprisingly, the mean yields increase by 4.19 and 8.09% ha^−1^ for ‘Salim’ and ‘Karim’, respectively. A significant variation of grain yield was recorded for both cultivars. In fact, the simulated yield ranged by 24–49 q ha^−1^ for ‘Salim’ and 18–34 q ha^−1^ for ‘Karim’. For the biomass, the mean simulated biomasses for both varieties over 51 years are about 121.68 q ha^−1^ (‘Salim’) and 48.47 q ha^−1^ (‘Karim’) (Figure 6), while the observed values were 100.19 q ha^−1^ and 44.66 q ha^−1^, respectively (Figure 5). The mean biomasses will therefore experience increases of 17.66% ha^−1^ for ‘Salim’ and 7.86% ha^−1^ for ‘Karim’. Long-term simulations have shown that the biomass of the ‘Salim’ cultivar ranged by 86–169 q ha^−1^ and ‘Karim’ by 32–80 q ha^−1^. Hence, this study revealed that the incidence of future climate events positively affects the yield of local durum wheat cultivars. According to the time slices (2016–2035, 2045–2064, and 2075–2094) and the agronomic scenarios considered, Bahri et al. [8] also mentioned a differential increase in durum wheat grain yield in the semi-arid region (Kef region), ranging from 5–11% under zero-tillage and 11–26% under zero-tillage with residue retention, as compared to the conventional tillage management. Additionally, a 0.2 °C temperature rise during 2020–2058 is expected to increase the yields (10.1–14.4% ha^−1^) and the biomass (13.6–14.6% ha^−1^) of three Tunisian barley cultivars [42]. However, the impact of a 1 °C temperature rise was only positive for the yield of the ‘Imen’ barley cultivar. In general, the main mechanisms controlling the simulated wheat responses are the direct and indirect temperature effects on the wheat’s phenological development. It is well known that warmer temperatures accelerate the plant growing cycle through advanced anthesis and maturity, thus resulting in smaller biomass accumulation and lower yields [7,43]. Several reports have noted the detrimental impact of extreme weather events and climate change on wheat productivity in the Mediterranean and Asian countries [44,45,46]. As predicted by Zhao et al. [25], wheat (6.0% reduction in global yields) will be the second affected species by each degree-Celsius increase in global mean temperature after maize (7.4%), while rice (3.2%) and soybean (3.1%) showed lower rates of increase. Nonetheless, an increase in atmospheric carbon dioxide (CO_2_) would have a fertilizing effect, especially when the temperature increase is less than 3 °C [47,48]. Middle and high latitudes, as shown in the Nebeur region (latitude: 36°17′), and rainfall changes will be beneficial to increased crop yields [40,49]. In addition, the presence of aerosols in the atmosphere (e.g., sulfur aerosol), limiting the rise in temperature, reduces the yields’ lowering but increases their variability [50]. This could thus rebalance the situation by limiting the harmful effect of a temperature rise. Rosenzweig and Tubiello [51] also concluded that the negative effects of temperature on simulated wheat yields might be reduced when minima increase more than maxima. However, in our case, the temperature minima decreased (−0.7 °C), while the maxima increased (2.3 °C), and precipitation showed an increase (Figure 6).

Different strategies may be adopted to reduce the risk of crop exposure to extreme events, including the choice of the genetic material. Considering the two durum wheat cultivars, ‘Salim’ yield potential was still much higher compared to that of ‘Karim’’, but the increase in grain yield is very close (1.51 and 1.89 q ha^−1^ for ‘Salim’ and ‘Karim’, respectively). This trend might be explained by the precocity of ‘Karim’ compared to ‘Salim’ (Table S2). Several authors pointed out that the use of wheat cultivars with a shorter growing cycle and early flowering can escape extreme climate events and thus result in a winning strategy for higher yields [7,52]. In contrast, Asseng et al. [53] indicated that a prolonged vegetative growth may better compensate for the reduced growing season length induced by higher temperatures. Otherwise, ‘Salim’ experienced a greater increase in biomass than ‘Karim’. Indeed, the ‘Salim’ cultivar is characterized by denser vegetation than ‘Karim’ (Table S2). In addition, ‘Salim’ showed an extended stay-green attribute compared to the control cultivar which might explain its better drought tolerance. In conclusion, the adoption of the ‘Salim’ cultivar associated with the recommended package could be a good strategy for adaptation to climate change. Given the significant role played by the extension and the access-related variables, increased emphasis on information dissemination, field demonstrations, the farmers’ participatory research, and training programs to popularize the new durum wheat cultivars and enhance their adoption rate are required. This also suggests that policy intervention should be made on improving the educational status of farming households and developing programs on a varietal package of durum wheat seed.

## 3. Materials and Methods

### 3.1. Site Description and Meteorological Data Collection

**Figure 7:**
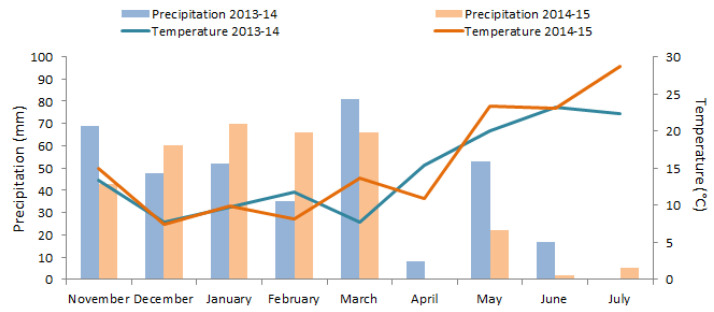
Monthly precipitation and temperature recorded in Nebeur/Kef site during 2013/14 and 2014/15 cropping seasons.

The experiments were conducted during two consecutive growing seasons, 2013/14 and 2014/15, in the Nebeur/Kef delegation (36°17′47″ N, 8°45′58″ E, at 390 m), located in the northwest of Tunisia. Before sowing, soil was collected over a 1 m depth and showed a sandy clay loam texture (59.7% sand, 26% clay, and 14.3% silt) [54], a cation exchange of 10 meq 100 g^−1^, and pH = 7.90. The growing season precipitation and temperature data were recorded at the meteorological station of the Kef region (Figure 7). The region is subject to a semi-arid climate. An observed daily series of the maximum and minimum air temperature, the precipitation, solar radiation, the maximum and minimum relative humidity, and the wind speed of the 28 years from 1992 to 2019 were also collected from the same station. During this period, the means of the maximum and minimum air temperature and precipitation were 38.0 °C, 11.8 °C, and 184 mm, respectively (Figure S1).

### 3.2. Experimental Procedures

Eleven farmers (leader and satellite farmers) were chosen in collaboration with Regional Agriculture Office Staff. The leader was selected based on his social relationship, his willingness to test and eventually to adopt new technologies, and his skill in communicating with the other farmers. All interventions (i.e., soil preparation, sowing, fertilization, weed and disease control, and harvesting) were conducted in the leader farmer’s field in the presence of the entire group (i.e., satellite farmers and extension agents). During the crop development, all the farmers monitored the incidence of diseases and pests and learned when they should apply chemicals. The aim of this experiment was an on-farm demonstration of durum wheat (*Triticum durum* Desf.) ‘Salim’ cultivar with an appropriate technical package using a participatory method. ‘Salim’, released in 2009 and marketed to farmers in 2019, is recognized as a drought-tolerant genotype (Table S2). This cultivar was compared to ‘Karim’, the most widely grown cultivar in Tunisia, released in 1980 but cultivated since 1973 (i.e., old cultivar). Each farmer (i.e., of the leader and satellite farmers) tested the two cultivars with the recommended package and the farmers’ usual practices. The difference between the demonstration package and the farmers’ practices is given in Table 1. During the two cropping seasons, 2013/14 and 2014/15, *Septoria tritici* blotch symptoms were developed in early April, in particular on ‘Karim’. Thus, Falcon (Tébuconazole + triadiménol+ spiroxamine) was recommended as a fungicide at a rate of 1 l ha^−1^ at the heading stage (Z51) [55]. Weeds were also chemically controlled using Puma^®^ evolution (fenoxaprop-*p*-ethyl + iodosulfuron-methyl sodium + mefenpyr-diethyl) (Bayer CropScience, Beja, Tunisia), at a rate of 1 l ha^−1^ at the 2–3 leaf stage (Z13).

### 3.3. Plant Measurements

At maturity, two agronomic traits were measured from the eleven field experiments: grain yield (q ha^−1^) and biomass (q ha^−1^). The analysis of variance (ANOVA) for each trait was performed using R statistical software version 4.0 (The R Foundation for Statistical Computing). The results obtained were used as a control to estimate the potential impact of climate change on the expected mean yield and the biomass by 2070, under the pedological conditions of the Nebeur region.

### 3.4. CropSyst Model Description

The crop growth model, CropSyst (version 3_04_08) was designed to determine the most appropriate form of durum wheat function response and its interaction with the surrounding environment and management [56]. This model operates at a daily time and the law of limiting factors (e.g., water, salt, and nitrogen stress) intervenes to determine the potential yield. Cropsyst was used for its ability to simulate durum wheat yields for several consecutive years and to establish the relationship between input and output for the durum wheat production with a range of management and weather scenarios [57]. The yield and biomass and their changes over time were analyzed.

### 3.5. Input Data and Weather Generation

As described by Stöckle and Nelson [58], the grain yield and biomass were simulated based on: (i) the simulation control (cultivar and technical package) based on field experiments; (ii) the soil file (soil texture, cation exchange capacity, and chemical characteristics) necessary to calculate the hydraulic properties and the soil water balance (runoff, infiltration, and evaporation) by the model; (iii) the position file (altitude, latitude, stiffness, slope length in addition to the climatic data [minimum and maximum temperature, precipitation, wind speed, minimum and maximum of relative humidity, and solar radiation of 28 years from 1992 to 2019]); (iv) the crop file describing the properties of the two durum wheat cultivars (e.g., GDD, degree day emergence, specific leaf area; Table S2); (v) and the management file including all the technical itineraries (e.g., sowing, fertilization, weed control; Table 1). The crop module includes the most sensitive parameters that are grouped by function, namely classification, sowing, growth, morphology, phenology, vernalization, photoperiod, dormancy, harvest, residues, nitrogen nutrition, sensitivity to salts, etc. The default values for a number of crop types are provided by Cropsyst and may need to be adjusted for local cultivars. Some parameters of the durum wheat cultivars were collected from the literature or estimated by specialists’ calculations.

Climate change, combined with other factors surrounding crops, might alter durum wheat conditions in the future; thus, daily weather series (2020–2070) were produced via the ClimGen weather generator of Cropsyst [57]. These series were derived from the observed weather parameters (1992–2019). We quote the main parameters (i.e., temperature and precipitation) on which this work is based. The released simulations were re-started each year after harvest using the soil water content for the 51 years of weather data (2020–2070) calculated using the initial soil water profile.

### 3.6. Short-Term Yield and Biomass Simulations

Following the work of collecting data relating to the durum wheat cultivars and package, we applied, in a first step, a crop-based model for each durum wheat cultivar combined with its package. Simulations were carried out short-term over two years, 2013/14 and 2014/15. This period was chosen based on the availability of the real yield and biomass for both durum wheat cultivars (‘Salim’ and ‘Karim’), which were considered as control data for the calibration and validation of the crop model. The first results obtained showed that the simulated yields approximated the observed yields for each cultivar with slight differences. These differences led us to act on certain physiological parameters of the wheat cultivars (growth, morphology, and phenology) to bring the simulated yields closer to the real yields. The model validation is a comparison between the outputs of the model and the perceived reality by an independent series of results. The validated short-term models were used for the long-term simulations over 51 years.

### 3.7. Long-Term Yield and Biomass Simulations

Once validated, the basic models were used for the long-term simulations, keeping the same cultivation techniques adopted by the farmers and using the climatic data generated from 2020 to 2070 to analyze the behavior and response of the two durum wheat cultivars, and the climate change impact on the yield and biomass.

## 4. Conclusions

This case study highlights the effect of the adoption of the ‘Salim’ cultivar and the recommended technical package on the grain yield and biomass. Interestingly, this cultivar performed better than the common cultivar, ‘Karim’. We also demonstrated that the use of ‘Salim’ with the proposed package markedly enhanced the yield and biomass compared to that of ‘Karim’ with the farmers’ usual practices. Otherwise, the generated climate change projections indicate +2.3 °C in maximum temperature, −0.7 °C in minimum temperature, and an increase of 160 mm in precipitation over the next 51 years (2021–2070). Surprisingly, this change positively affected the yield and biomass of ‘Salim’ conducted with the new technical package. Subsequently, this adoption could be an alternative to mitigate climate change impacts in future scenarios for the Nebeur region. Such results ultimately help farmers to develop more profit-oriented behaviors, which are necessary to enhance adoption rate, production, and food security in the long term. This strategy could be applied to formulate spatially targeted adaptation methods to large areas of North Africa with similar environmental characteristics for sustainable farming systems.

## Figures and Tables

**Table 1:** Technical itineraries used in demonstration plots and farmers’ plots during 2013/14 and 2014/15 cropping seasons.

Cultural Operation	Growth Stage	Technical Package	Farmers’ Practices
Sowing date	-	Last week of November	Last week of November
Rate of sowing		160 kg ha^−1^	160 kg ha^−1^
Sowing depth		1.2 cm	1.2 cm
Weed control	2–3 leaf stage (Z13)	Weed control	No weed control
Disease control	Heading stage (Z51)	Disease control	No disease control
Fertilization	At sowing	100 kg ha^−1^ Di-ammonium phosphate (18% N and 46% P_2_O_5_)	100 kg ha^−1^ Di-ammonium phosphate (18% N and 46% P_2_O_5_)
	3–4 leaf stage (Z14)	100 kg ha^−1^ ammonium nitrate (N 33.5%)	-
	Beginning of tillering (Z21)	100 kg ha^−1^ ammonium nitrate (N 33.5%)	100 kg ha^−1^ ammonium (N 33.5%)
	Heading stage (Z51)	150 kg ha^−1^ ammonium nitrate (N 33.5%)	-
Irrigation	-	Rainfed	

## Data Availability

Available upon reasonable request.

## Supplementary Materials

The following are available online at https://www.mdpi.com/article/10.3390/plants10112379/s1: Figure S1—monthly precipitation (**a**) and temperature (**b**) recorded in Nebeur/Kef site during 1992–2019, Table S1—quantification of on-farm yield gap for all farmers, Table S2—characteristics of selected durum wheat cultivars, ‘Salim’ and ‘Karim’.
