# Supplementary material for: Adoption of Durum Wheat Cultivar ‘Salim’ with a Technical Package and Its Resilience to Climate Change Impacts in Smallholders: Case of Nebeur/Kef Region, Tunisia
Source: Plants (Basel). 2021 Nov 5;10(11):2379. doi: 10.3390/plants10112379 (PMC8623015; doi:10.3390/plants10112379)
Supplement: Supplementary file 1 [file plants-10-02379-s001.zip › plants-1405902-supplementary.pdf]

Supplementary Materials

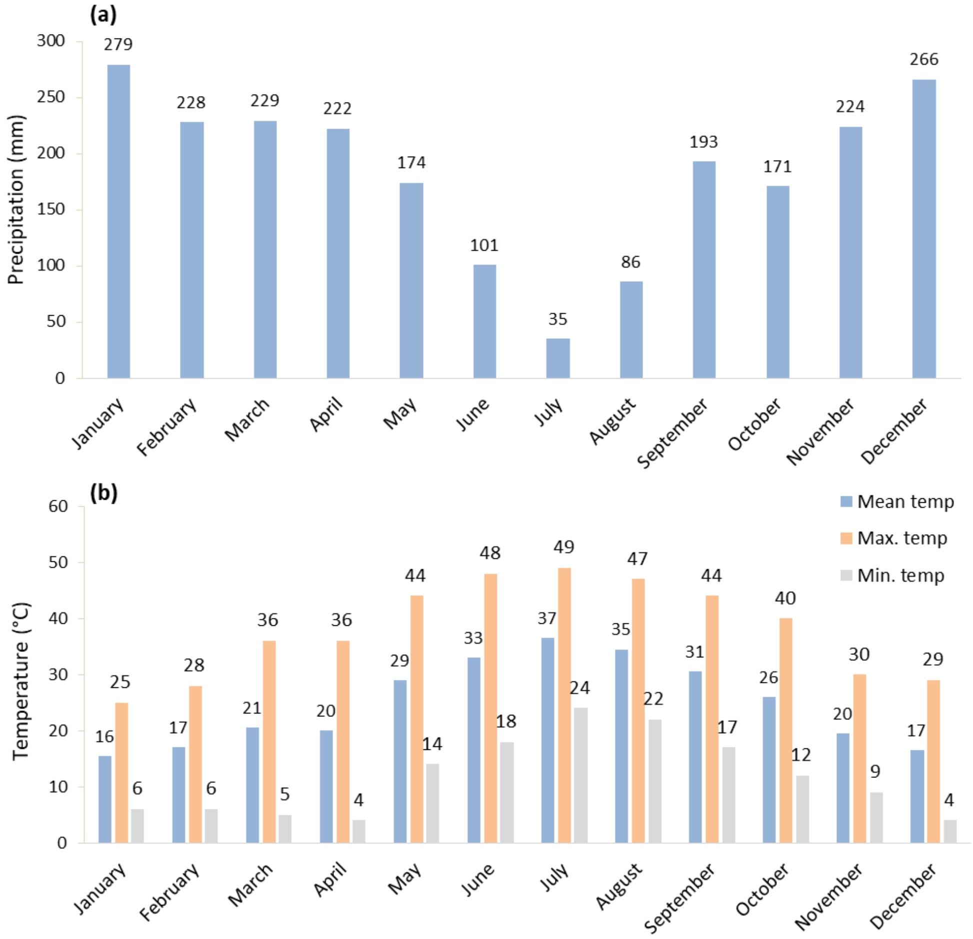

Figure S1. Monthly precipitation (**a**) and temperature (**b**) recorded in Nebeur/Kef site during 1992-2019.

Table S1. Quantification of on-farm yield gap for all farmers.

Farmers		Yield gap (q ha^{-1})	
		2013/14	2014/15
1	Leader	12	12
2	Satellite	10	9
3	Satellite	13	11
4	Satellite	15	14
5	Satellite	16	15
6	Satellite	15	12
7	Satellite	13	12
8	Satellite	14	13
9	Satellite	14	13
10	Satellite	15	12
11	Satellite	15	13
Mean		13.8	12.3

Table S2. Characteristics of selected durum wheat cultivars, 'Salim' and 'Karim'.

Parameters	Salim	Karim
Seed	Certified	Certified
Degree day emergence (°C-days)	122	117
Specific leaf area (cm^2)	27	24
GDD (°C day from planting to heading)	1223.85	1208.10
Mean Evatranspiration	Penman Montieth	
Salinity tolerance exponent	0.9	1.48
Phenologic sensitivity to water stress	0.97	1.01
Susceptibility to diseases	More resistant to powdery mildew, rust (brown and yellow) and septoria than Karim	Susceptible to disease mainly septoria, rusts, etc.
Unstressed harvest index	0.28	0.36
Productivity	Very productive	Productive
Thousand kernel weight (g)	40-48	40-45
